# Supplementary material for: 2.5 Å-resolution structure of human CDK-activating kinase bound to the clinical inhibitor ICEC0942
Source: Biophys J. 2021 Jan 19;120(4):677–86. doi: 10.1016/j.bpj.2020.12.030 (PMC7896097; doi:10.1016/j.bpj.2020.12.030)
Supplement: Document S1. Fig. S1–S4 [file mmc1.pdf]

Biophysical Journal, Volume *120*

Supplemental Information

2.5 Å-resolution structure of human CDK-activating kinase bound to the clinical inhibitor ICEC0942

Basil J. Greber, Jonathan Remis, Simak Ali, and Eva Nogales

A

B

C

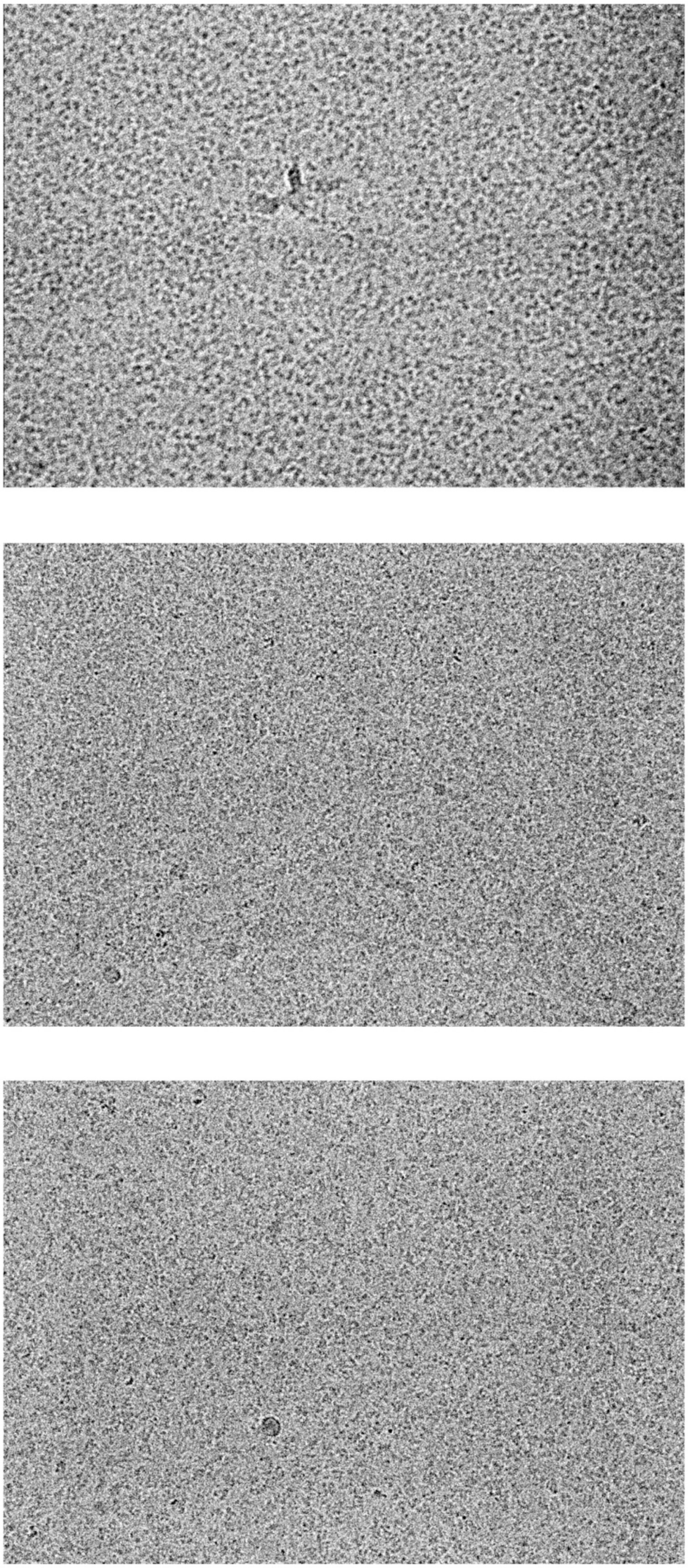

Figure S1. Example micrographs. (**A**) Drift-corrected and summed movie acquired during grid screening at high defocus (approx. 3 µm underfocus). The high defocus helps visualising the distribution of CAK particles in vitreous ice. (**B**, **C**) Drift-corrected and summed movies acquired during data collection for high-resolution structure determination of CAK-ICEC0942 (B, grid 2; C, grid 3) at approx. 1.1-1.2 µm underfocus. The contrast in all three panels has been enhanced to visualise the particles, which are otherwise hard to see due to the low image contrast inherent in cryo-EM data.

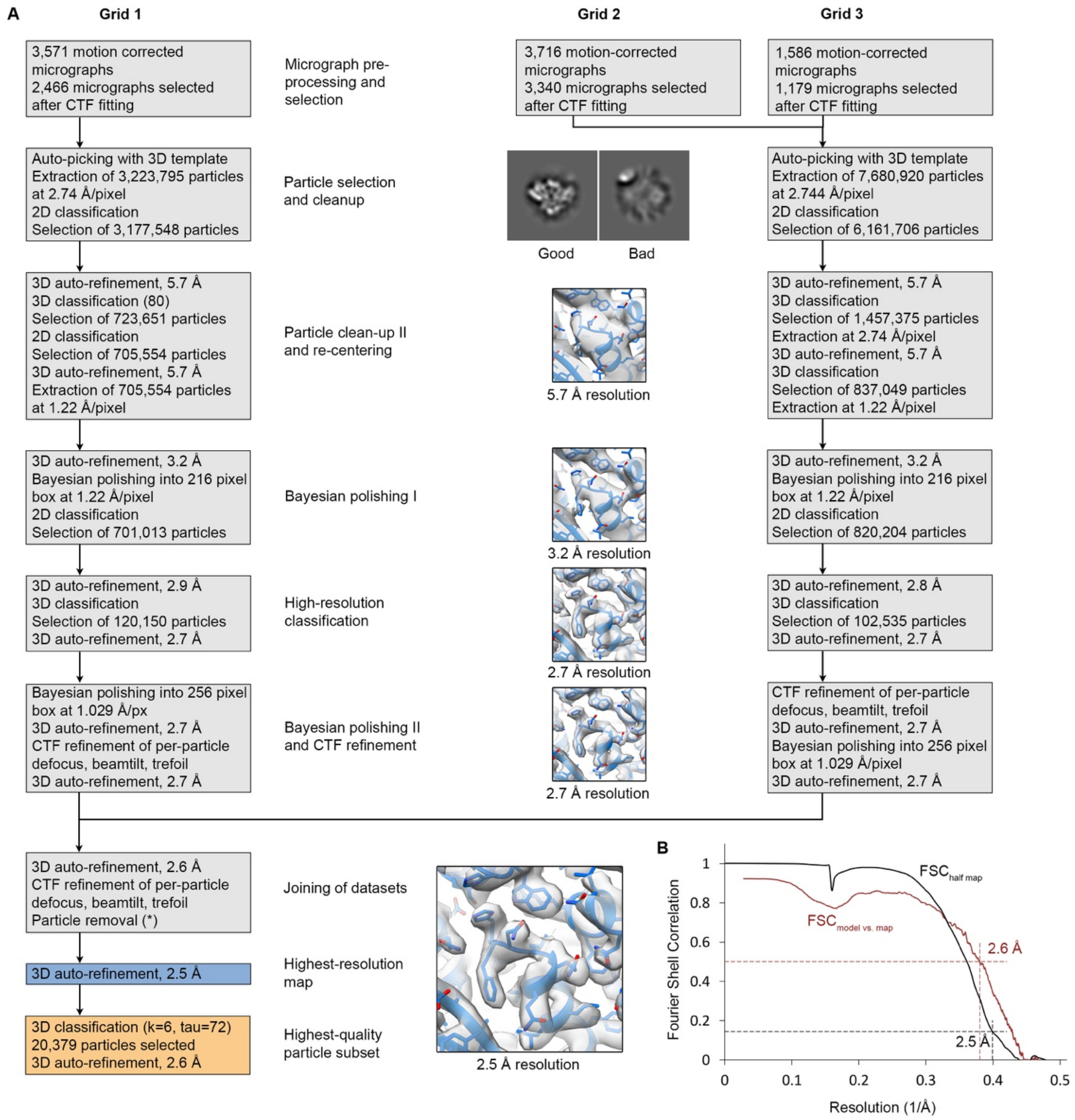

Figure S2. Data processing strategy. (**A**) Individual processing steps are listed, and major phases of processing are outlined. Densities are RELION refinement output maps, shown without B-factor sharpening applied. At the particle removal step (*), particles that came from micrographs with worse than 4 Å estimated CTF fit resolution as well as a very small number of duplicated particles were removed. (**B**) Fourier shell correlation curve computed between cryo-EM half-maps (black) and the map and the refined model (red). Estimated resolutions are indicated.

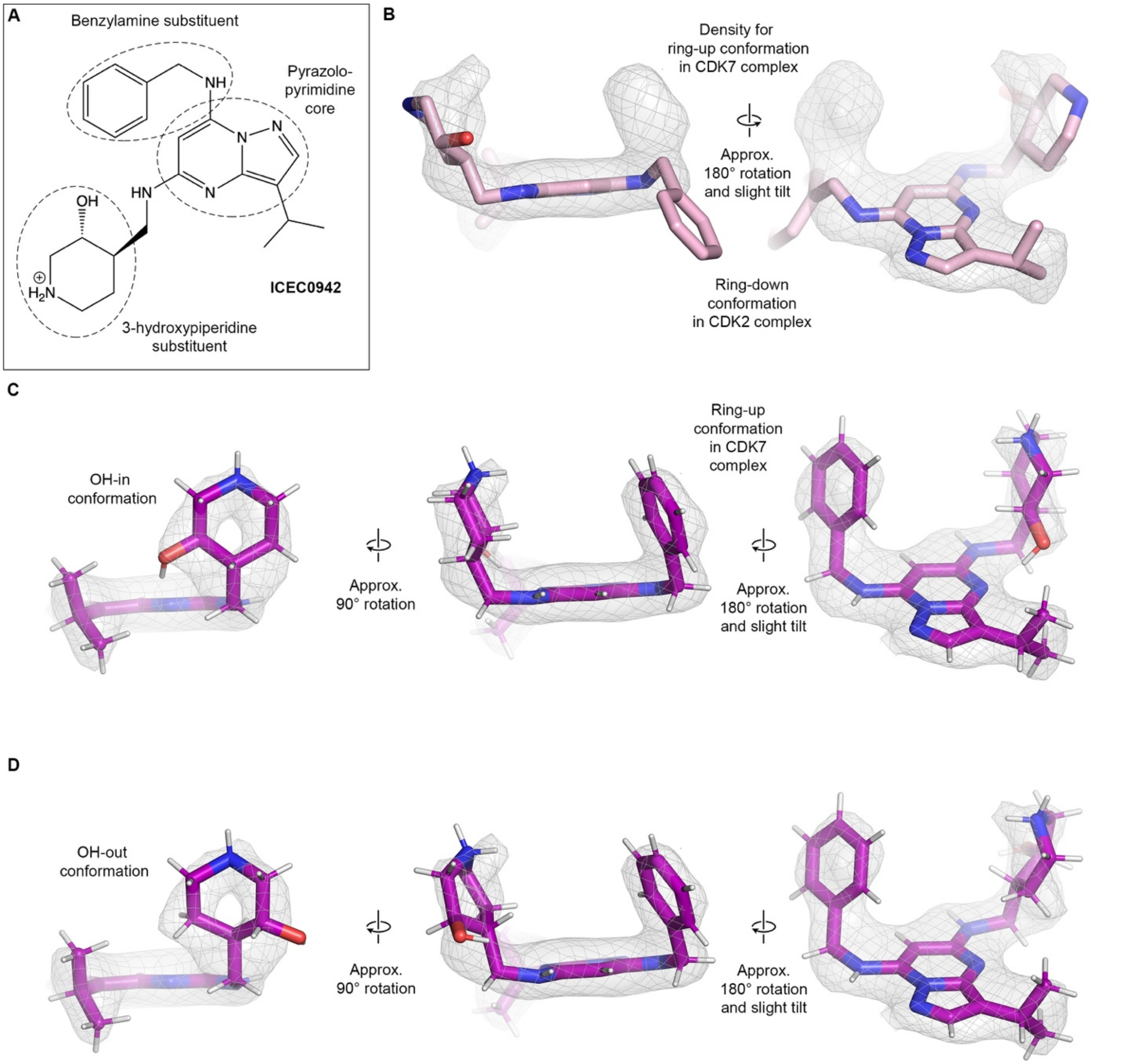

Figure S3. Ligand fitting. (**A**) Chemical structure of ICEC0942. Chemical groups discussed in the text are labelled. (**B**) Comparison of the density for ICEC0942 in complex with CDK7 (density grey) and the structure of the inhibitor as observed bound to CDK2 (pink). The two views are related by a rotation of approx. 180°, with the right hand-side view slightly tilted forward for best visibility of the map fit. (**C**, **D**) Views of ICEC0942 (purple) in the OH-in (C) and OH-out conformations (D) after refinement with PHENIX-OPLS3e, shown with the cryo-EM density (grey). The middle and right-hand side view are the same as in (B). The view on the left is related to the middle panels by a rotation of approx. 90°.

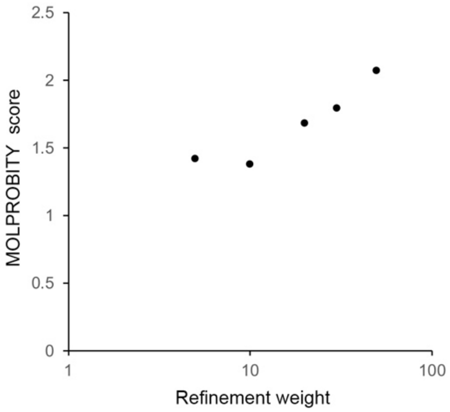

Figure S4. PHENIX-OPLS3e refinement. Due to the higher bond length and bond angle R.M.S.D induced by the use of the OPLS3e force field, an explicit scan across refinement weights in PHENIX was conducted instead of using automated weight determination. Higher weights lead to better fit to the map but worse structure quality as measured by the MOLPROBITY score. We chose 10 as the optimum weight for refinement.
