# Supplementary material for: 2.5 Å-resolution structure of human CDK-activating kinase bound to the clinical inhibitor ICEC0942
Source: Biophys J. 2021 Jan 19;120(4):677–86. doi: 10.1016/j.bpj.2020.12.030 (PMC7896097; doi:10.1016/j.bpj.2020.12.030)
Supplement: Document S2. Article plus supporting material [file mmc2.pdf]

Biophysical Journal
Article

Biophysical Society

2.5 Å-resolution structure of human CDK-activating kinase bound to the clinical inhibitor ICEC0942

Basil J. Greber,[1,2,3,*] Jonathan Remis,[2] Simak Ali,[4] and Eva Nogales[2,3,5,6]
[1]Division of Structural Biology, The Institute of Cancer Research, London, United Kingdom; [2]California Institute for Quantitative Biosciences (QB3), University of California, Berkeley, Berkeley, California; [3]Molecular Biophysics and Integrative Bio-Imaging Division, Lawrence Berkeley National Laboratory, Berkeley, California; [4]Division of Cancer, Department of Surgery & Cancer, Imperial College London, London, United Kingdom; [5]Department of Molecular and Cell Biology, University of California, Berkeley, Berkeley, California and [6]Howard Hughes Medical Institute, University of California, Berkeley, Berkeley, California

ABSTRACT The human CDK-activating kinase (CAK), composed of CDK7, cyclin H, and MAT1, is involved in the control of transcription initiation and the cell cycle. Because of these activities, it has been identified as a promising target for cancer chemotherapy. A number of CDK7 inhibitors have entered clinical trials, among them ICEC0942 (also known as CT7001). Structural information can aid in improving the affinity and specificity of such drugs or drug candidates, reducing side effects in patients. Here, we have determined the structure of the human CAK in complex with ICEC0942 at 2.5 Å-resolution using cryogenic electron microscopy. Our structure reveals conformational differences of ICEC0942 compared with previous X-ray crystal structures of the CDK2-bound complex, and highlights the critical ability of cryogenic electron microscopy to resolve structures of drug-bound protein complexes without the need to crystalize the protein target.

SIGNIFICANCE Detailed knowledge of the three-dimensional structure of biological macromolecules provides mechanistic insight into their function and aids structure-based drug design efforts. Such applications require high-resolution structures, determination of which has remained challenging for small biomolecules (small proteins, nucleic acids, or their complexes) that do not readily crystallize. Here, we use cryogenic electron microscopy to determine the structure of the human CDK-activating kinase complex bound to ICEC0942, an inhibitor of CDK7 that is undergoing clinical trials. This work paves the way toward development of next-generation CDK7 inhibitors with higher effectivity and/or specificity and explores the ability of cryogenic electron microscopy to visualize small asymmetric biomolecules at high resolution.

INTRODUCTION

The human cyclin-dependent kinase (CDK)-activating kinase (CAK) is a trimeric complex comprising CDK7 as well as cyclin H and MAT1 (1,2). CDK7 and cyclin H form a typical CDK-cyclin pair (1), whereas MAT1 is a CAK assembly factor (3,4) that also regulates CDK7 activity (3,5) and attaches the CAK to transcription factor IIH (TFIIH) to promote phosphorylation of the C-terminal heptapeptide repeat domain of the RNA polymerase II subunit RPB1 (5,6). In addition to its role in RNA polymerase II phosphorylation, the human CAK also phosphorylates numerous other targets involved in transcription and the cell cycle (7), including other CDKs (1). Because of their dual function in regulation of transcription and the cell cycle, CDK7 and the CAK are important regulators of cell growth and cell division. Deregulation of these pathways leads to human disease, including cancer, and many cancers rely on aberrantly upregulated transcription to sustain their growth and proliferation (8). Therefore, CDK7 has been identified as a promising drug target for cancer treatment (9). Numerous compounds that inhibit CDK7 have been developed, and several of them are currently undergoing clinical trials (10). Among those clinical CDK7 inhibitors is the pyrazolopyrimidine derivative ICEC0942 (also known as CT7001) (11), which binds CDK7 noncovalently but with high affinity and selectivity for CDK7 over other CDKs.

High-resolution three-dimensional (3D) structures of drug targets and drug-bound complexes provide insight into the molecular basis of drug binding and allow structure-guided design and optimization of the next generation of drug

Submitted November 5, 2020, and accepted for publication December 21, 2020.

*Correspondence: basil.greber@icr.ac.uk

Editor: Bridget Carragher.
https://doi.org/10.1016/j.bpj.2020.12.030

© 2021 Biophysical Society.
This is an open access article under the CC BY license (http://creativecommons.org/licenses/by/4.0/).

candidates. However, structural data are currently lacking for CDK7 bound to inhibitors in clinical trials, including ICEC0942. Traditionally, such information has come from X-ray crystallographic experiments, but not all drug targets can be readily crystallized. In the past few years, cryogenic electron microscopy (cryo-EM) has revolutionized structural biology, allowing structure determination of biological macromolecules that are difficult to crystallize (12). Cryo-EM structures beyond 2-Å resolution have been obtained for a few large or symmetric complexes (13–18). However, many drug targets are neither large nor symmetric. Despite substantial progress, such as on the study of G protein-coupled receptors with data collected on a 300-kV electron microscope with energy filter (19), the high-resolution structure determination of asymmetric complexes below 100-kDa molecular weight at 2.5-Å or better resolution has generally remained more challenging.

The human CAK is a representative of the class of important drug targets that are not easily crystallized, and which still constitute a challenge for cryo-EM because they are asymmetric and of relatively small size, with the CDK-cyclin module of the CAK having a molecular mass of only ~85 kDa. Structures of human CAK and of the fungal homolog TFIIK, both with nucleotide ligands, have been determined recently at 2.8-Å resolution using cryo-EM (20) and at 2.6-Å using X-ray crystallography (21), whereas a human CAK complex modified by the covalently bound inhibitor THZ1 was resolved at only 3.3 Å (20). Using a 200-kV cryo-transmission electron microscope without an energy filter, we have now determined the structure of the human CAK-ICEC0942 complex at 2.5 Å, a resolution that allowed docking of ICEC0942 and analysis of the molecular interactions that are formed between the drug and its target. We also analyzed key statistics in our data set to estimate the feasibility of even higher-resolution reconstructions in the future.

MATERIALS AND METHODS

Protein expression, purification, and complex formation

Full-length wild-type CAK was expressed in insect cells and purified as previously described (20). For complex formation, CAK was diluted 5× in cryo-EM buffer (20 mM HEPES-KOH (pH 7.9), 200 mM KCl, 2 mM $MgCl_2$, and 5 mM β-mercaptoethanol) from a stock concentration of ~2 mg/mL and incubated with 50 μM ICEC0942 (dissolved in water at a stock concentration of 5 mM) for 5 min at room temperature. ICEC0942 was synthesized as previously described (11).

Cryo-EM grid preparation

UltrAuFoil 1.2/1.3 gold foil grids (Quantifoil Micro Tools, Grosslobichau, Germany) were plasma cleaned for 30–45 s using a Tergeo plasma cleaning device (PIE Scientific, Union City, CA). 4 μL of the complex were applied to the grid, immediately blotted for 6–7 s using a Vitrobot Mark IV (Thermo Fisher Scientific, Waltham, MA), and flash frozen in a mixture of ethane and propane (22).

Data collection

Grids were transferred into autogrid cassettes and loaded into a Talos Arctica cryo-transmission electron microscope (Thermo Fisher Scientific). Data were acquired with the microscope set at 200-kV acceleration voltage and 72,886-fold magnification, resulting in a pixel size of 0.686 Å on the object scale. Cryo-electron micrograph movies of 70 frames per movie were recorded using a K3 direct electron detector camera (Gatan, Pleasanton, CA) in super-resolution mode with a total electron exposure of 69 $e^- Å^{-2}$, resulting from a 2 s exposure at ~34 $e^- Å^{-2} sec^{-1}$. A total of 3571, 3716, and 1586 micrographs were collected from three different grids. The data collection was supervised using on-the-fly processing in cryoSPARC Live (Structura Biotechnology, Toronto, Canada; https://cryosparc.com/live) (23) to monitor microscope performance, micrograph quality, and orientation distribution of the particles on the grid. Representative sample micrographs are shown in Fig. S1.

Image processing

Image processing was performed according to the strategy previously described for the structure determination of human CAK (20) and as outlined in Fig. S2. Initial processing occurred in two batches: one batch comprising the data from grid 1, the second batch comprising the data from grids 2 and 3. Image processing was performed in RELION 3.1 (24), unless stated otherwise. Super-resolution electron micrograph movies were aligned using MotionCor2 (25), within RELION 3.1, or using the CPU implementation of motion correction within RELION 3.1 (24) with 2× binning. Contrast transfer function (CTF) estimation was performed using CTFFIND 4.1 (26) from within RELION. Micrographs and CTF fits were inspected to remove poor-quality micrographs, resulting in the selection of 2466 micrographs from grid 1, 3340 micrographs from grid 2, and 1179 micrographs from grid 3. Particles were picked using template-based autopicking in RELION 3.1 and the published cryo-EM map of human CAK as in Greber et al. (20). Particles were initially extracted in 256 × 256-pixel boxes (extraction boxes are given in physical detector pixels, not super-resolution pixels) and downscaled to 64 × 64 pixels for faster processing. Initial two-dimensional (2D) classification served mostly to eliminate false-positive picks or ice contamination (Fig. S2 *A*); other classes were included very liberally, and this step did not serve to preselect high-quality particles at this stage. The selected particles were then cleaned up further by alignment-free 3D classification after 3D autorefinement, in the case of the data from grid 1, complemented by another 2D classification step (Fig. S2 *A*). After re-extraction and scaling to 144 × 144 pixels (1.220 Å/pixel), the selected particles were again autorefined, and the data sets reached ~3.2-Å resolution at this stage. During the first round of Bayesian polishing (27), applied to more than 700,000 particles from each data set, the particle boxes were enlarged to 384 × 384 pixels (with subsequent downscaling to 216 × 216 pixels, 1.220 Å/pixel) to retain the high-resolution signal delocalized by the CTF (28). Because the enlargement of the box may lead to inclusion of artifacts or edges in the reboxed particles, one round of 2D classification was carried out to eliminate any particles affected by such issues. After this procedure, the data sets were refined to higher resolution (2.8- to 2.9-Å resolution), 3D classified without alignment, then subjected to another round of Bayesian polishing and to CTF refinement (29). During polishing, the 384 × 384-pixel extraction box size was retained, and particles were downscaled to 256 × 256 pixels at a pixel size of 1.029 Å. Individual data sets reached 2.7-Å resolution after subsequent autorefinement. The two data sets were joined, autorefined, CTF refined, and autorefined again to reach a final resolution of 2.5 Å at Fourier shell correlation (FSC) = 0.143 (Fig. S2 *B*). The refinement was conducted following the gold-standard strategy, and FSC thresholds were chosen accordingly (30). The map was isotropic, with a sphericity value of 0.95 as computed by the 3D FSC validation server (31). The output map was sharpened by application of a B-factor of −45 $Å^2$, automatically estimated in RELION, and low-pass filtered according to the half-map FSC curve.

Identification of a higher-quality particle subset

The 205,478-particle data set was subjected to alignment-free 3D classification using six classes and a τ-value of 72. The resulting classes were refined. One class reaching 2.6-Å resolution from 20,379 particles was used for further analysis (henceforth termed the higher-quality subset). It is important to note that, in our case, the remaining classes also refined to ~2.6- to 2.8-Å resolution (with ~60,000 and 20,000 particles needed for the higher and lower ends of that spectrum, respectively), indicating that the quality within the 205,478-particle data set was already high in general. Notably, these reconstructions were near-identical to each other and did not show meaningful conformational differences.

For the comparisons between data subsets (i.e., between the 205,478-particle data set that achieved 2.5 Å-resolution and the higher-quality 20,379-particle subset that achieved 2.6-Å resolution), the corresponding data parameters were extracted from the refinement star file generated by RELION. For the CTF fit resolution and the maximal value of the probability distribution, these values could be used directly. The defocus values used are the average of the two defocus parameters determined for each micrograph (as required to account for astigmatism). Because of the extremely large sample size (one data point for each particle in the two data sets) and the fact that ever-smaller differences become statistically significant as the sample size increases (32), all comparisons across the two data subsets (CTF fit resolution, maximal value of the probability distribution, and defocus values) show a statistically significant difference when analyzed using significance tests. However, the effect size in the case of CTF fit resolution is so small (difference between subset mean values = 0.04 Å) that it is meaningless in practice (i.e., for data analysis or micrograph selection).

Refinement of random subsets

Random subsets were generated using the corresponding functionality in RELION 3.1 and re-refined using complete global alignment search from a reference filtered to 20-Å resolution. Masks and solvent-corrected FSCs were used during all refinements but are particularly important for the very small particle subsets.

Model building and initial refinement

Our previous structure of the human CAK (Protein Data Bank, PDB: 6XBZ) (20) was docked into the density and rebuilt in Coot (33). The rebuilding was aided by maps processed using LAFTER (34) or subjected to density modification in PHENIX (35,36) using the program phenix.resolve_cryo_em (37), in which the positions of protein backbone carbonyls could be deduced in some regions. The ligand model and corresponding refinement restraints were generated in phenix.elbow (38), docked into the density, and adjusted. After completion of protein rebuilding and ligand fitting, water molecules were placed into the remaining unassigned densities near the protein. To avoid false placements, we limited water placement to densities with σ-levels of 3.5 root mean-squared deviations above background or higher. We verified that interpretation of these densities as water molecules was justified by comparing our water placements to the waters built in the X-ray crystal structures of cyclin H at 2.6-Å resolution (PDB: 1KXU, 1JKW) (39,40) and found a substantial degree of agreement between these entirely independent placements. The structure was initially refined using phenix.real_space_refine (35).

Refinement using PHENIX-OPLS3e

We subsequently employed a combination of PHENIX with the OPLS3e force field, described recently (41), to more accurately refine the OH-in and OH-out conformations of ICEC0942 (Fig. S3). As previously described (41), we performed an explicit weight scan (Fig. S4) using refinement weights ranging from 5 to 50 and selected a refinement weight of 10, which gave the optimum MolProbity score, for the final PHENIX-OPLS3e refinement to generate the structure used for interpretation.

As reported previously (41), the approach employing the OPLS3e force field results in an improved overall MolProbity score and lower clash scores at the expense of higher bond and angle root mean-squared deviation values (Table 1). To facilitate comparison of the quality and refinement statistics of our higher-resolution CAK structure with results obtained by methods that are more routinely used in the field, the coordinate model was also refined with PHENIX alone, omitting OPLS3e. For this refinement, the ligand was restrained to the conformations resulting from the PHENIX-OPLS3e refinement using reference restraints. The refinement statistics of this model are excellent according to commonly used standards, with no Ramachandran or rotamer outliers (Table 1). However, it is worth noting the following: 1) there are instances in which the PHENIX-OPLS3e-refined side chain conformations appear to be more accurate representations of the density and 2) in the absence of restraints to preserve the ligand to the conformation found using OPLS3e, PHENIX refines the hydroxypiperidine ring into an energetically less favorable boat conformation that also appears to fit the map less well. We thus conclude that the use of PHENIX-OPLS3e provides useful additional structural detail at the resolution of our cryo-EM map.

All structures were validated using MolProbity (42) as implemented within PHENIX, including the recently introduced Rama-Z measure (43). The refinement statistics for the coordinate models are given in Table 1.

Accession codes

The 2.5 Å-resolution cryo-EM map and atomic coordinates of the CAK-ICEC0942 complex have been deposited in the EMDataResource (EMD) and the PDB with accession codes EMD: 12042 and PDB: 7B5O (PHENIX) and PDB: 7B5Q (PHENIX-OPLS3e), respectively. The 2.6-Å map has been deposited with accession code EMD: 11823, and the 3.0-Å resolution map from 5000 particles has been deposited with accession code EMD: 11828. Original micrograph videos have been deposited in the Electron Microscopy Public Image Archive database with accession code EMPIAR: 10561.

RESULTS AND DISCUSSION

Structure determination

To obtain insight into the mode of binding of ICEC0942 (11) to the human CAK, we collected cryo-EM data of the CAK-ICEC0942 complex using a 200-kV electron microscope equipped with a direct detector capable of electron counting, as used previously for structure determination of the CAK (20) and other small complexes (44). Our previous cryo-EM visualization of human CAK indicated that preferred orientation is an issue with this specimen and that the orientation distribution of the complex on the grid can vary substantially, even between grids prepared using near-identical conditions. This was the case with the CAK-ICEC0942 complex as well, with pronounced peaks for top and side views on different grids that had been prepared within minutes of each other using the same materials (Fig. 1 *A*). Therefore, we monitored the data collections using cryoSPARC Live (23) and proceeded with 1- or 2-day automated data collection only after verifying that the grid being imaged was able to provide a sufficient range of views to result in a near-isotropic 3D reconstruction that was then

TABLE 1 Data collection and refinement statistics

Data set	CAK-ICEC0942			
Microscope	Talos Arctica			
Stage type	Autoloader			
Voltage (kV)	200			
Detector	Gatan K3			
Acquisition mode	super-resolution			
Physical pixel size (Å)	0.686			
Defocus range (μm)	0.3–1.6			
Electron exposure (e^-/A^2)	69			
Reconstruction	EMD: 12042		EMD: 11823	EMD: 11828
Software	RELION 3.1		RELION 3.1	RELION 3.1
Particles picked	10,904,715		10,904,715	10,904,715
Particles final	205,478		20,397	5000
Extraction box size (pixels)	384 × 384 × 384		384 × 384 × 384	384 × 384 × 384
Rescaled box size (pixels)	256 × 256 × 256		256 × 256 × 256	256 × 256 × 256
Final pixel size (Å)	1.029		1.029	1.029
Accuracy rotations (°)	0.80		0.70	0.88
Accuracy translations (Å)	0.25		0.21	0.31
Map resolution (Å)	2.5		2.6	3.0
Map resolution range	2.4–2.8		2.6–2.9	NA
Map sharpening B-factor ($Å^2$)	−45		23	−20
Coordinate refinement				
Software and algorithm	PHENIX	PHENIX-OPLS3e	NA	NA
Clipped box size (pixels)	128	128	NA	NA
Resolution cutoff (Å)	2.51	2.51	NA	NA
$FSC_{model\text{-}versus\text{-}map} = 0.5$ (Å)	2.6	2.6	NA	NA
Model	PDB: 7B5O	PDB: 7B5Q	NA	NA
Number of residues	715	715	NA	NA
Protein	642	642	NA	NA
Ligand (ICEC0942/H_2O)	1/73	1/73	NA	NA
B-factors overall	39.3	42.4	NA	NA
Protein	39.4	42.6	NA	NA
Ligand (ICEC0942/H_2O)	33.8/31.9	39.8/37.3	NA	NA
R.M.S. deviations				
Bond lengths (Å)	0.007	0.091	NA	NA
Bond angles (°)	0.717	2.403	NA	NA
Validation				
MolProbity score	1.66	1.37	NA	NA
MolProbity clashscore	8.72	2.67	NA	NA
Rotamer outliers (%)	0.0	1.25	NA	NA
C_β deviations (%)	0.0	0.82	NA	NA
Ramachandran plot				
Favored (%)	96.7	96.3	NA	NA
Allowed (%)	3.3	3.0	NA	NA
Outliers (%)	0.0	0.6	NA	NA
Ramachandran *Z*-scores				
Whole	0.0	−1.8	NA	NA
Helix	0.6	−1.0	NA	NA
Sheet	−0.4	0.1	NA	NA
Loop	−0.8	−1.4	NA	NA

NA, not applicable.

further improved by combining data sets from three grids (Fig. 1 *A*).

From a total of 6985 selected micrographs, 10,904,715 particles were picked. A homogeneous subset of 205,478 particles was identified by 2D and 3D image classification (Fig. S2 *A*) and then refined to 2.5 Å resolution employing recent data analysis tools, such as Bayesian polishing and correction of optical aberrations in silico (Fig. 1 *B*; Fig. S2 *B*; Materials and methods; (24,27,29)). The very large number of initially selected particles and the ability to subsequently obtain a highly homogeneous and self-consistent particle population by stringent 3D classification likely contributed to the high resolution achieved. Our structure at this improved resolution (Fig. 2 *A*) is highly consistent with our previous structures of CAK-ATPγS at 2.8 Å and CAK-THZ1 at 3.3-Å resolution (20), showing the complex in the same structural state, with an active conformation of the regulatory T-loop and the inhibitor bound to

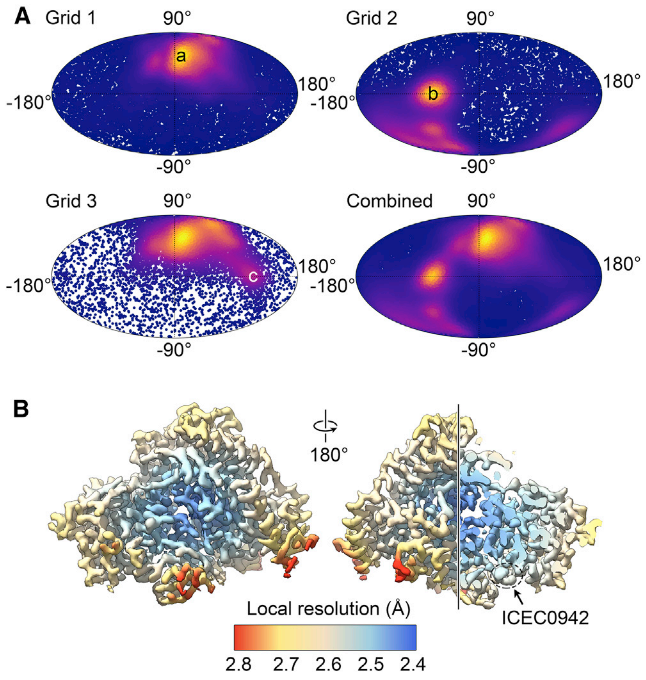

FIGURE 1 Data collection high-resolution reconstruction. (*A*) Mollweide plots are given, showing the orientation distribution of the particles from the three grids used for structure determination of the CAK-ICEC0942 complex. Two main maxima of preferred orientation are visible, labeled a (*side view*) and b (*bottom view*). The weak maximum on grid 3 (labeled c) is the same view as b after rotation of the particle by 180°. (*B*) Local resolution estimate is shown, indicating up to 2.4-Å resolution in the core of CAK (part of the complex is cut away to reveal the core and ICEC0942 in the *right-hand panel*) and ~2.5- to 2.6-Å resolution at the site where ICEC0942 binds (indicated).

the active site of the kinase (Fig. 2 *A*). The use of the higher-resolution map in combination with density modification in PHENIX (37) allowed us to improve and correct the backbone geometry in some well-resolved areas, showing density features corresponding to backbone carbonyls (Fig. 2, *B* and *C*). The density for the inhibitor is clear and shows a hole in the 3-hydroxypiperidine group within ICEC0942 (Fig. 2, *D* and *E*), probably because the carbon-carbon single bonds in this six-membered ring are longer than the partial double bonds in aromatic ring systems, which generally do not show holes at this resolution.

Insight into ICEC0942 binding

Analysis of the conformation of ICEC0942 in the active site pocket of CDK7 revealed that the pyrazolopyrimidine core of the inhibitor (Fig. S3 *A*) binds in a very similar position and orientation as previously observed for CDK2-bound ICEC0942 (45), forming hydrogen bonds with both the backbone carbonyl and the amide proton of CDK7 residue M94 (Fig. 2, *D* and *E*). However, we find conformational differences of both of the six-ring-bearing substituents of the inhibitor compared with the CDK2-bound structure (Fig. 2 *F*; Fig. S3 *B*).

The orientation of the benzylamine substituent in CDK7-bound ICEC0942 differs substantially from the conformation of this substituent in the CDK2-ICEC0942 complex (45). The two conformations ("ring-up" and "ring-down"; Fig. S3 *B*) are related by a rotation of this chemical group by ~120° (Fig. 2 *F*; Fig. S3 *B*). In the ring-up conformation, the benzyl group is accommodated by the linker between the N- and C-terminal domains of CDK7 rather than forming interactions with the β-sheet near the N-terminus of the kinase, as observed in the ring-down conformation in CDK2. This conformational difference is likely due to two subtle structural differences between CDK7 and CDK2. First, the substitution of residue I10 in CDK2 by L18 in CDK7 is likely to cause one of the terminal methyl groups in CDK7 L18 to interfere with inhibitor in the ring-down conformation (Fig. 2 *F*). Second, a slight shift of the protein backbone near the C-terminus of the interdomain linker (around CDK2 L83/CDK7 M94) moves CDK2 residue D86 toward the ICEC0942 binding site. This conformational arrangement would probably lead to clashes between the inhibitor in the ring-up conformation and the side chain of D86 in CDK2 (Fig. 2 *F*), thus disfavoring this inhibitor conformation.

A second conformational difference between the CDK7- and CDK2-bound structures involves the 3-hydroxypiperidine ring of ICEC0942. The density shows that, in the CDK7 complex, this ring is tilted forward slightly (Fig. S3 *B*). The protonated secondary amine of the hydroxypiperidine substituent remains within hydrogen bonding distance of the side-chain amide oxygen of CDK7 N142 (Fig. 2 *D*). The features of the cryo-EM density additionally suggest that the hydroxypiperidine ring may be rotated by ~180° in the CDK7-bound structure (Fig. S3 *C*), such that the hydroxy group points toward the inside of the active site cavity in CDK7 (OH-in conformation), whereas it points toward the solvent and a neighboring β-strand in CDK2 (OH-out; Fig. 2, *D* and *F*; Fig. S3, *B* and *D*). To enable a more accurate refinement of the ligand and a more detailed analysis of these interactions, we refined our structure using a recently described method combining the OPLS3e force field with the PHENIX refinement package (41). Using this approach, we were able to refine both the OH-in and OH-out conformations into the cryo-EM density. Our density is most compatible with the OH-in orientation, but the OH-out conformation may be present simultaneously and was modeled as an alternative conformer (Fig. S3, *C* and *D*).

The origin of the conformational change in the hydroxypiperidine ring of CDK7-bound ICEC0942 relative to CDK2 is not entirely clear but might be connected to small conformational differences at the base of the T-loop near the active site, where CDK7 residue D155 is moved toward this region of the inhibitor slightly, with the Cβ atom most closely approaching the hydroxypiperidine ring. The basis of this hypothesis is that our CAK-ICEC0942 structure contains MAT1 and cyclin H in addition to CDK7, which stabilize the extended, active, conformation of the T-loop (20). In

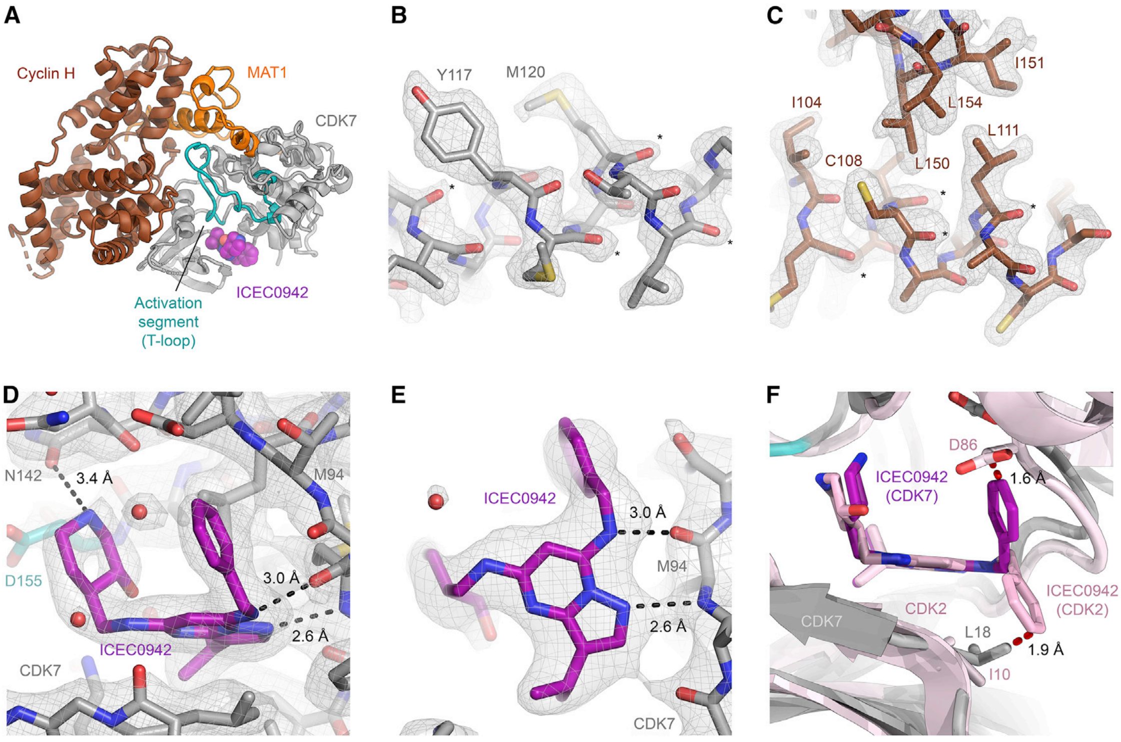

FIGURE 2 Structure of the human CAK-ICEC0942 complex. (*A*) Overview of the structure, with cyclin H shown in brown, MAT1 in orange, and CDK7 in gray (T-loop *teal*, ICEC0942 *purple*). (*B* and *C*) The map after density modification in PHENIX is shown. Notable side chains are labeled, and features for backbone carbonyls are indicated by asterisks. Density-modified maps as shown in these panels were used for visualization only. Maps postprocessed in RELION 3.1 (24) without density modification were used for coordinate refinement. (*D* and *E*) Fit of ICEC0942 (only the OH-in conformation is shown for clarity) in the cryo-EM map showing views of the hydroxypiperidine ring of ICEC0942 (*D*) and the pyrazolopyrimidine core of ICEC0942 (*E*), with likely hydrogen bonds indicated. The map shown resulted from standard postprocessing in RELION 3.1. Modeled water molecules are represented by red spheres. (*F*) Comparison of the CDK7-ICEC0942 and CDK2-ICEC0942 (*pink*) structures. The benzylamine substituent assumes distinct conformations in the two structures. Prohibitively close contacts (2 Å or less) that disfavor the alternative conformations in CDK2 and CDK7 are indicated in red.

contrast, the CDK2-ICEC0942 structure (45) was solved using isolated CDK2, which results in an inactive T-loop conformation. The conformational differences between the active and inactive states are most dramatic at the tip of the T-loop, but smaller changes extend to the base of the T-loop near the active site cleft, where they might affect the ICEC0942 binding site.

Previous molecular dynamics analysis identified the G-rich loop as well as residues D137 and D155 of CDK7 as possible additional interaction partners of the hydroxypiperidine ring on ICEC0942 (45). We do not observe direct hydrogen bonding between these elements in our structure. As already observed in the molecular dynamics simulation (45), D137 lies relatively deep inside the active site pocket and may be inaccessible without larger motions of the inhibitor, which might require the breaking of other interactions, thus rendering such conformations rarely accessed. CDK7 D155 (shown in Fig. 2 *D*), the mutation of which to alanine has been shown to negatively affect inhibitor binding (45), lies within possible hydrogen bonding distance of the CDK2-like conformation of ICEC0942 but not of the conformation we observe within CDK7. It is possible, however, that D155 stabilizes the side-chain conformation of N142, which in turn interacts with ICEC0942 (Fig. 2 *D*), providing a possible explanation of the experimentally observed contribution of the carboxyl group of D155 to ICEC0942 binding. Any direct (rather than water-mediated) interactions between ICEC0942 and the G-rich loop of CDK7 would require some conformational dynamics of either the protein or the inhibitor because our structure shows the G-rich loop slightly removed from the active site pocket, beyond the reach of direct interactions, and at a somewhat lower density contour, indicating flexibility.

These conformational differences and differing interactions between the CDK7- and CDK2-bound ICEC0942 (Fig. 2 *F*) may provide avenues to the design of more specific inhibitors and drug candidates with reduced side effects.

Data quality and limitations

Previous efforts to determine the cryo-EM structure of small complexes have shown that high-resolution reconstructions can be obtained from very small numbers (a few tens of

thousands) of asymmetric units selected by classification of larger data sets (44,46). We therefore analyzed the resolutions that could be achieved using particle subsets randomly extracted from our 2.5 Å-resolution data set, ranging from 50,000 particles down to 1500 particles (Fig. 3 *A*). Independently, we 3D-subclassified our 2.5 Å-resolution data set and identified a small class of 20,397 particles that could be refined to 2.6-Å resolution. Even though these particles could not match the 2.5 Å-resolution obtained using the 205,478-particle data set, they arguably represent a higher-quality subset that results in only a minor decrease in resolution with a 10-fold smaller particle number. We then refined random subsets of the smaller, higher-quality class as well. These calculations revealed, as expected under the assumption that these particles represent a higher-quality subclass, that the Henderson-Rosenthal B-factor (30) for this smaller subclass is lower, and that random data subsets from this class containing as few as 5000 particles are sufficient to reach 3.0-Å resolution (Fig. 3, *B* and *C*). This number of asymmetric units is less than half of that used in an asymmetric reconstruction of alcohol dehydrogenase to 3.1-Å resolution (~11,000 particles) (44).

To investigate possible differences between these 20,397 higher-quality particles and those comprising the larger 205,478-particle data set that might guide future data collection or processing efforts, we compared the defocus values, the resolution to which the CTF fit agrees with the power spectrum of the micrograph (as a measure of micrograph quality), and the maximal value of the probability function in the orientation search (which correlates with the sharpness of the orientation assignment) between the larger set and the smaller subset of particles. The analysis shows

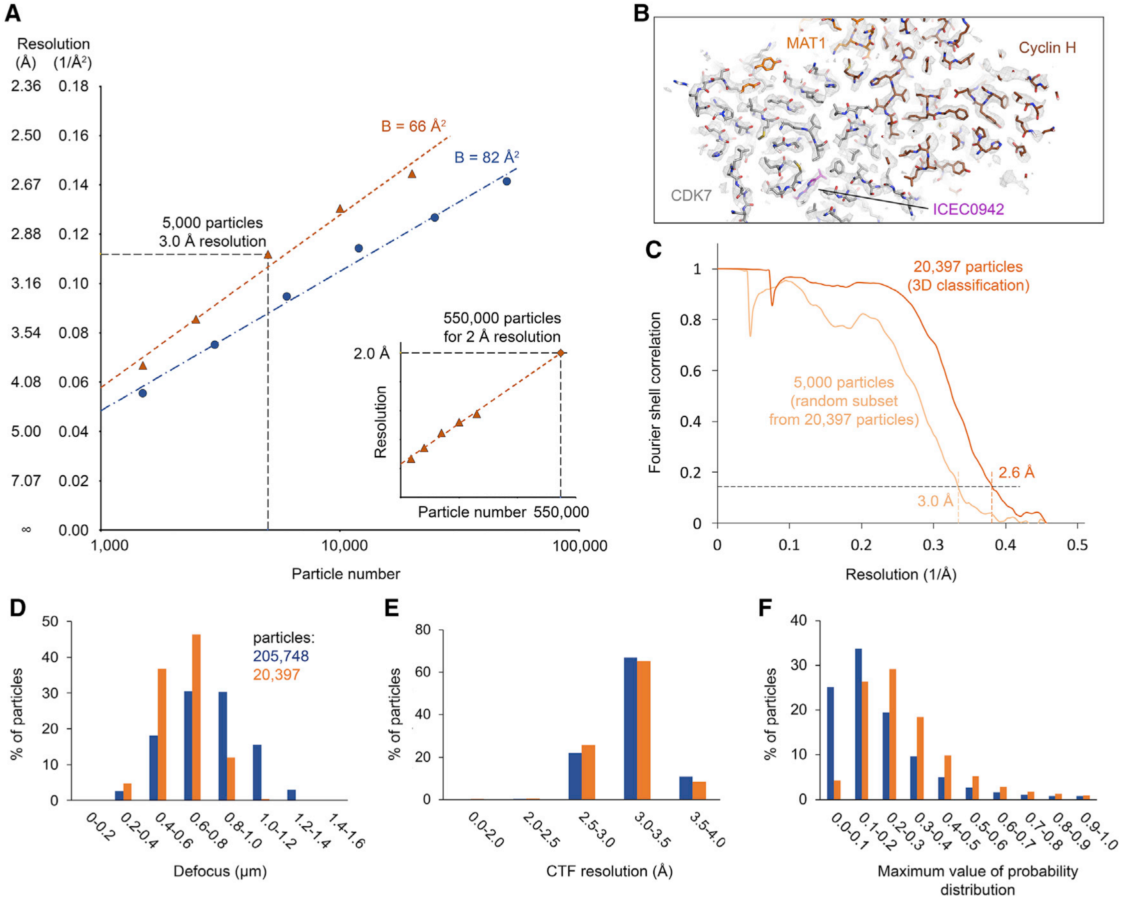

FIGURE 3 Analysis of data quality and achievable resolution. (*A*) Henderson-Rosenthal plot (30) graphing the inverse-squared resolution against the logarithm of the number for reconstructions from data subsets randomly picked from the full 205,478-particle data set (*blue*) and the 20,397-particle subclass (*yellow*). 5000 randomly selected particles from the latter data set were still sufficient to generate a 3.0-Å resolution structure. B-factors were computed from the slope of a linear fit through the data points and are indicated. Inset: Extrapolation to higher particle number and higher resolution, assuming a linear relationship between the logarithm of the particle number and the inverse-squared resolution. (*B*) Density from a 3.0-Å resolution reconstruction from 5000 random particles from the 20,397-particle data set. The density is readily interpretable, despite slightly elevated noise levels due to the small number of particles. (*C*) FSC curves for the reconstruction from the 20,397-particle subclass and the 3.0-Å reconstruction of 5000 particles randomly selected from that subclass. (*D*–*F*) Comparison of micrograph and particle properties between the 205,478-particle data set and the higher-quality 20,397-particle subclass. Particles from the higher-quality subclass were collected at lower average defocus (*D*) and exhibited higher maximal values of the probability function in the orientation search (*F*) but showed very little difference in the quality of the initial CTF fitting (*E*).

that the smaller data set is enriched in lower-defocus particles, with mean defocus and standard deviations of 0.79 ($\sigma = 0.22$) μm and 0.63 ($\sigma = 0.14$) μm for the full set and the subset, respectively (Fig. 3 *D*), in agreement with the notion that low-defocus data collection is beneficial (19). In light of recent data demonstrating that higher defocus does not induce information loss due to a CTF envelope when using modern electron microscopes with highly coherent field emission guns (47), the source of this effect in our data set is unclear but may be related to the data processing strategy. Signal delocalization at higher defocus (28) can limit the achievable resolution when small particle boxes are used and might lead to elimination of high-defocus particles during high-resolution 3D classification. However, even though the delocalized 2.5-Å signal can be recovered at defocus values of 1.3 μm or lower at the center of an image box of size of 263 Å, particles with defocus values of 0.8–1.0 μm, which should not suffer from adverse effects of signal delocalization, were substantially underrepresented after our final 3D classification along with all higher-defocus particles. This indicates that effects other than signal delocalization may have contributed to the result we observe. It is possible that the faster oscillations of the high-frequency CTF at higher defocus lead to larger high-resolution phase errors for higher-defocus data in the presence of small errors in the estimated defocus values that are likely unavoidable, even with in silico CTF refinement.

We also saw that the estimated resolution to which the initially fitted CTF agrees with the power spectrum of the micrograph, a metric that is often used to assess micrograph quality, is essentially the same between the two particle sets, with mean values of 3.18 ($\sigma = 0.26$) Å and 3.14 ($\sigma = 0.26$) Å for the full set and the subset, respectively (Fig. 3 *E*). We note that we had removed a small number of particles from all micrographs not reaching 4 Å according to this metric during data processing. However, particles from micrographs clearing this modest threshold generally appear to be able to contribute to the high-quality reconstruction. Analysis of the beam-induced motion in the first few video frames also showed no difference between the two data subsets (data not shown). Finally, particles in the smaller subset show higher maximal values of the probability distribution describing the angle assignment, with median values of 0.17 and 0.26 for the full set and the subset, respectively, indicating that these particles were more accurately aligned (Fig. 3 *F*).

It is worth noting that the results from particle classification were better than those obtained by simply removing particles with higher defocus or with broader probability distributions from the data set. For example, removing all particles with a maximal value of the probability distribution for the angular assignment of less than 0.25 resulted in a data set of 57,473 particles that refined to only 2.8-Å resolution, and removing all particles with a defocus above 0.8 μm yielded 89,562 particles that refined to 2.7-Å resolution (data not shown). Both of these compare unfavorably with the 2.6 Å obtained from 20,397 3D-classified particles. This suggests that approaches that exploit particle classification to identify high-quality particles may be preferable to applying stringent selection criteria to the micrographs at the outset of data processing, at least for these metrics.

Extrapolation of the particle number required to reach higher resolution shows that almost 30-fold more particles are required to reach 2-Å resolution, even in the best case (i.e., when assuming linearity of the extrapolation (Fig. 3 *A*, *inset*)), which may not hold true in practice. Clearly, this is not feasible by simply collecting more of the same data, and an increase in either data quality or particle yield is needed. Higher data quality could likely be achieved using improved microscope hardware, as recently described for record-breaking apo-ferritin structures (16,18), or by increasing the magnification to achieve higher detective quantum efficiency (DQE) at a given spatial frequency (13), with the consequent need to collect more images to obtain the same number of particles. Alternatively, in the light of the extremely low yield of "good" particles of ~0.2–2% (20,397 and 205,478 particles in the final reconstructions from 9,339,254 particles after initial 2D classification), it will be critical to increase the yield of intact, high-quality particles while producing better orientation distributions. Improved sample preparation using new instrumentation (48,49), specimen support films that minimize the interaction of the particles with the air-water interface (46,50,51), or grids with substantially reduced beam-induced motion (52) should allow cryo-EM structure determination at higher resolution from smaller or equal amounts of sample and without additional microscope hardware, both of which may aid in reducing the overall cost of the experiment.

CONCLUSIONS

Here, we have presented the structure of a small asymmetric protein complex, the human CAK, with a cancer drug candidate bound in its active site cleft. Our structure reveals differences between CDK2- and CDK7-bound ICEC0942, highlighting the importance of obtaining structural insight into the interactions of drugs or other ligands with their native protein targets in their physiological complexes that are not limited by the availability of diffracting crystals. Future efforts should be directed toward establishing methods that can routinely resolve small, asymmetric complexes to beyond 2-Å resolution. This would allow direct and reliable visualization of the protein backbone carbonyl conformation and of the majority of ordered water molecules, thus providing more accurate insight into hydrogen bonding networks.

SUPPORTING MATERIAL

Supporting material can be found online at https://doi.org/10.1016/j.bpj.2020.12.030.

AUTHOR CONTRIBUTIONS

B.J.G. and S.A. designed the study. S.A. provided ICEC0942. B.J.G. and J.R. collected the cryogenic electron microscopy data. B.J.G. processed the cryogenic electron microscopy data, built the molecular model, and interpreted the structure with input from E.N. and S.A. B.J.G. wrote the initial draft of the study, and all authors contributed to the final version.

ACKNOWLEDGMENTS

We thank P. Grob and A. Chintangal for support with electron microscopy and computing, respectively, and J. Fraser and S. Wankowicz for discussions on ligand fitting methods. Data were collected at Cal-Cryo at the California Institute for Quantitative Biosciences (QB3) of the University of California Berkeley.

This work was supported by National Institute of General Medical Sciences grant R35GM127018 to E.N. B.J.G. was supported by an Institute of Cancer Research Fellowship (grant GFR151X). E.N. is a Howard Hughes Medical Investigator. S.A. is funded by Cancer Research UK grant C37/A18784. S.A. is a named inventor on CDK7 inhibitor patents and has shares in Carrick Therapeutics.

REFERENCES

1. Fisher, R. P., and D. O. Morgan. 1994. A novel cyclin associates with MO15/CDK7 to form the CDK-activating kinase. *Cell.* 78:713–724.
2. Devault, A., A. M. Martinez, …, M. Dorée. 1995. MAT1 (‘menage à trois’) a new RING finger protein subunit stabilizing cyclin H-cdk7 complexes in starfish and Xenopus CAK. *EMBO J.* 14:5027–5036.
3. Fisher, R. P., P. Jin, …, D. O. Morgan. 1995. Alternative mechanisms of CAK assembly require an assembly factor or an activating kinase. *Cell.* 83:47–57.
4. Tassan, J. P., M. Jaquenoud, …, E. A. Nigg. 1995. In vitro assembly of a functional human CDK7-cyclin H complex requires MAT1, a novel 36 kDa RING finger protein. *EMBO J.* 14:5608–5617.
5. Busso, D., A. Keriel, …, J. M. Egly. 2000. Distinct regions of MAT1 regulate cdk7 kinase and TFIIH transcription activities. *J. Biol. Chem.* 275:22815–22823.
6. Rossignol, M., I. Kolb-Cheynel, and J. M. Egly. 1997. Substrate specificity of the cdk-activating kinase (CAK) is altered upon association with TFIIH. *EMBO J.* 16:1628–1637.
7. Rimel, J. K., Z. C. Poss, …, D. J. Taatjes. 2020. Selective inhibition of CDK7 reveals high-confidence targets and new models for TFIIH function in transcription. *Genes Dev.* 34:1452–1473.
8. Bradner, J. E., D. Hnisz, and R. A. Young. 2017. Transcriptional addiction in cancer. *Cell.* 168:629–643.
9. Fisher, R. P. 2019. Cdk7: a kinase at the core of transcription and in the crosshairs of cancer drug discovery. *Transcription.* 10:47–56.
10. Sava, G. P., H. Fan, …, S. Ali. 2020. CDK7 inhibitors as anticancer drugs. *Cancer Metastasis Rev.* 39:805–823.
11. Patel, H., M. Periyasamy, …, S. Ali. 2018. ICEC0942, an orally bioavailable selective inhibitor of CDK7 for cancer treatment. *Mol. Cancer Ther.* 17:1156–1166.
12. Nogales, E., and S. H. W. Scheres. 2015. Cryo-EM: a unique tool for the visualization of macromolecular complexity. *Mol. Cell.* 58:677–689.
13. Wu, M., G. C. Lander, and M. A. Herzik, Jr. 2020. Sub-2 Angstrom resolution structure determination using single-particle cryo-EM at 200 keV. *J Struct Biol X.* 4:100020.
14. Tan, Y. Z., S. Aiyer, …, D. Lyumkis. 2018. Sub-2 Å Ewald curvature corrected structure of an AAV2 capsid variant. *Nat. Commun.* 9:3628.
15. Merk, A., T. Fukumura, …, S. Subramaniam. 2020. 1.8 Å resolution structure of β-galactosidase with a 200 kV CRYO ARM electron microscope. *IUCrJ.* 7:639–643.
16. Yip, K. M., N. Fischer, …, H. Stark. 2020. Atomic-resolution protein structure determination by cryo-EM. *Nature.* 587:157–161.
17. Watson, Z. L., F. R. Ward, …, J. H. Cate. 2020. Structure of the bacterial ribosome at 2 Å resolution. *eLife.* 9:e60482.
18. Nakane, T., A. Kotecha, …, S. H. W. Scheres. 2020. Single-particle cryo-EM at atomic resolution. *Nature.* 587:152–156.
19. Danev, R., M. Belousoff, …, P. M. Sexton. 2020. Routine sub-2.5 Å cryo-EM structure determination of B-family G protein-coupled receptors. *bioRxiv* https://doi.org/10.1101/2020.08.21.260851.
20. Greber, B. J., J. M. Perez-Bertoldi, …, E. Nogales. 2020. The cryoelectron microscopy structure of the human CDK-activating kinase. *Proc. Natl. Acad. Sci. USA.* 117:22849–22857.
21. Peissert, S., A. Schlosser, …, C. Kisker. 2020. Structural basis for CDK7 activation by MAT1 and Cyclin H. *Proc. Natl. Acad. Sci. USA.* 117:26739–26748.
22. Tivol, W. F., A. Briegel, and G. J. Jensen. 2008. An improved cryogen for plunge freezing. *Microsc. Microanal.* 14:375–379.
23. Punjani, A., J. L. Rubinstein, …, M. A. Brubaker. 2017. cryoSPARC: algorithms for rapid unsupervised cryo-EM structure determination. *Nat. Methods.* 14:290–296.
24. Zivanov, J., T. Nakane, …, S. H. Scheres. 2018. New tools for automated high-resolution cryo-EM structure determination in RELION-3. *eLife.* 7:e42166.
25. Zheng, S. Q., E. Palovcak, …, D. A. Agard. 2017. MotionCor2: anisotropic correction of beam-induced motion for improved cryo-electron microscopy. *Nat. Methods.* 14:331–332.
26. Rohou, A., and N. Grigorieff. 2015. CTFFIND4: fast and accurate defocus estimation from electron micrographs. *J. Struct. Biol.* 192:216–221.
27. Zivanov, J., T. Nakane, and S. H. W. Scheres. 2019. A Bayesian approach to beam-induced motion correction in cryo-EM single-particle analysis. *IUCrJ.* 6:5–17.
28. Sigworth, F. J. 2016. Principles of cryo-EM single-particle image processing. *Microscopy (Oxf.).* 65:57–67.
29. Zivanov, J., T. Nakane, and S. H. W. Scheres. 2020. Estimation of high-order aberrations and anisotropic magnification from cryo-EM data sets in *RELION*-3.1. *IUCrJ.* 7:253–267.
30. Rosenthal, P. B., and R. Henderson. 2003. Optimal determination of particle orientation, absolute hand, and contrast loss in single-particle electron cryomicroscopy. *J. Mol. Biol.* 333:721–745.
31. Tan, Y. Z., P. R. Baldwin, …, D. Lyumkis. 2017. Addressing preferred specimen orientation in single-particle cryo-EM through tilting. *Nat. Methods.* 14:793–796.
32. Sullivan, G. M., and R. Feinn. 2012. Using effect size-or why the P value is not enough. *J. Grad. Med. Educ.* 4:279–282.
33. Emsley, P., B. Lohkamp, …, K. Cowtan. 2010. Features and development of coot. *Acta Crystallogr. D Biol. Crystallogr.* 66:486–501.
34. Ramlaul, K., C. M. Palmer, and C. H. S. Aylett. 2019. A local agreement filtering algorithm for transmission EM reconstructions. *J. Struct. Biol.* 205:30–40.
35. Afonine, P. V., B. K. Poon, …, P. D. Adams. 2018. Real-space refinement in PHENIX for cryo-EM and crystallography. *Acta Crystallogr. D Struct. Biol.* 74:531–544.
36. Adams, P. D., P. V. Afonine, …, P. H. Zwart. 2010. PHENIX: a comprehensive Python-based system for macromolecular structure solution. *Acta Crystallogr. D Biol. Crystallogr.* 66:213–221.
37. Terwilliger, T. C., O. V. Sobolev, …, R. J. Read. 2020. Density modification of cryo-EM maps. *Acta Crystallogr. D Struct. Biol.* 76:912–925.
38. Moriarty, N. W., R. W. Grosse-Kunstleve, and P. D. Adams. 2009. Electronic Ligand Builder and Optimization Workbench (eLBOW): a tool

for ligand coordinate and restraint generation. *Acta Crystallogr. D Biol. Crystallogr.* 65:1074–1080.

39. Andersen, G., A. Poterszman, …, J. C. Thierry. 1996. The crystal structure of human cyclin H. *FEBS Lett.* 397:65–69.

40. Kim, K. K., H. M. Chamberlin, …, S. H. Kim. 1996. Three-dimensional structure of human cyclin H, a positive regulator of the CDK-activating kinase. *Nat. Struct. Biol.* 3:849–855.

41. van Zundert, G. C. P., N. W. Moriarty, …, K. W. Borrelli. 2020. Macromolecular refinement of X-ray and cryo-electron microscopy structures with Phenix/OPLS3e for improved structure and ligand quality. *bioRxiv* https://doi.org/10.1101/2020.07.10.198093.

42. Williams, C. J., J. J. Headd, …, D. C. Richardson. 2018. MolProbity: more and better reference data for improved all-atom structure validation. *Protein Sci.* 27:293–315.

43. Sobolev, O. V., P. V. Afonine, …, P. D. Adams. 2020. A global ramachandran score identifies protein structures with unlikely stereochemistry. *Structure.* 28:1249–1258.e2.

44. Herzik, M. A., Jr., M. Wu, and G. C. Lander. 2019. High-resolution structure determination of sub-100 kDa complexes using conventional cryo-EM. *Nat. Commun.* 10:1032.

45. Hazel, P., S. H. B. Kroll, …, P. S. Freemont. 2017. Inhibitor selectivity for cyclin-dependent Kinase 7: a structural, thermodynamic, and modelling study. *ChemMedChem.* 12:372–380.

46. Han, Y., X. Fan, …, N. Yan. 2020. High-yield monolayer graphene grids for near-atomic resolution cryoelectron microscopy. *Proc. Natl. Acad. Sci. USA.* 117:1009–1014.

47. Glaeser, R. M., W. J. H. Hagen, …, C. J. Russo. 2020. Defocus-dependent thon-ring fading. *bioRxiv* https://doi.org/10.1101/2020.10.12.336214.

48. Jain, T., P. Sheehan, …, C. S. Potter. 2012. Spotiton: a prototype for an integrated inkjet dispense and vitrification system for cryo-TEM. *J. Struct. Biol.* 179:68–75.

49. Klebl, D. P., M. S. C. Gravett, …, S. P. Muench. 2020. Need for speed: examining protein behavior during CryoEM grid preparation at different timescales. *Structure.* 28:1238–1248.e4.

50. Han, B.-G., Z. Watson, …, R. M. Glaeser. 2016. Long shelf-life streptavidin support-films suitable for electron microscopy of biological macromolecules. *J. Struct. Biol.* 195:238–244.

51. D'Imprima, E., D. Floris, …, W. Kühlbrandt. 2019. Protein denaturation at the air-water interface and how to prevent it. *eLife.* 8:e42747.

52. Naydenova, K., P. Jia, and C. J. Russo. 2020. Cryo-EM with sub-1 Å specimen movement. *Science.* 370:223–226.

Biophysical Journal, Volume *120*

Supplemental Information

2.5 Å-resolution structure of human CDK-activating kinase bound to the clinical inhibitor ICEC0942

Basil J. Greber, Jonathan Remis, Simak Ali, and Eva Nogales

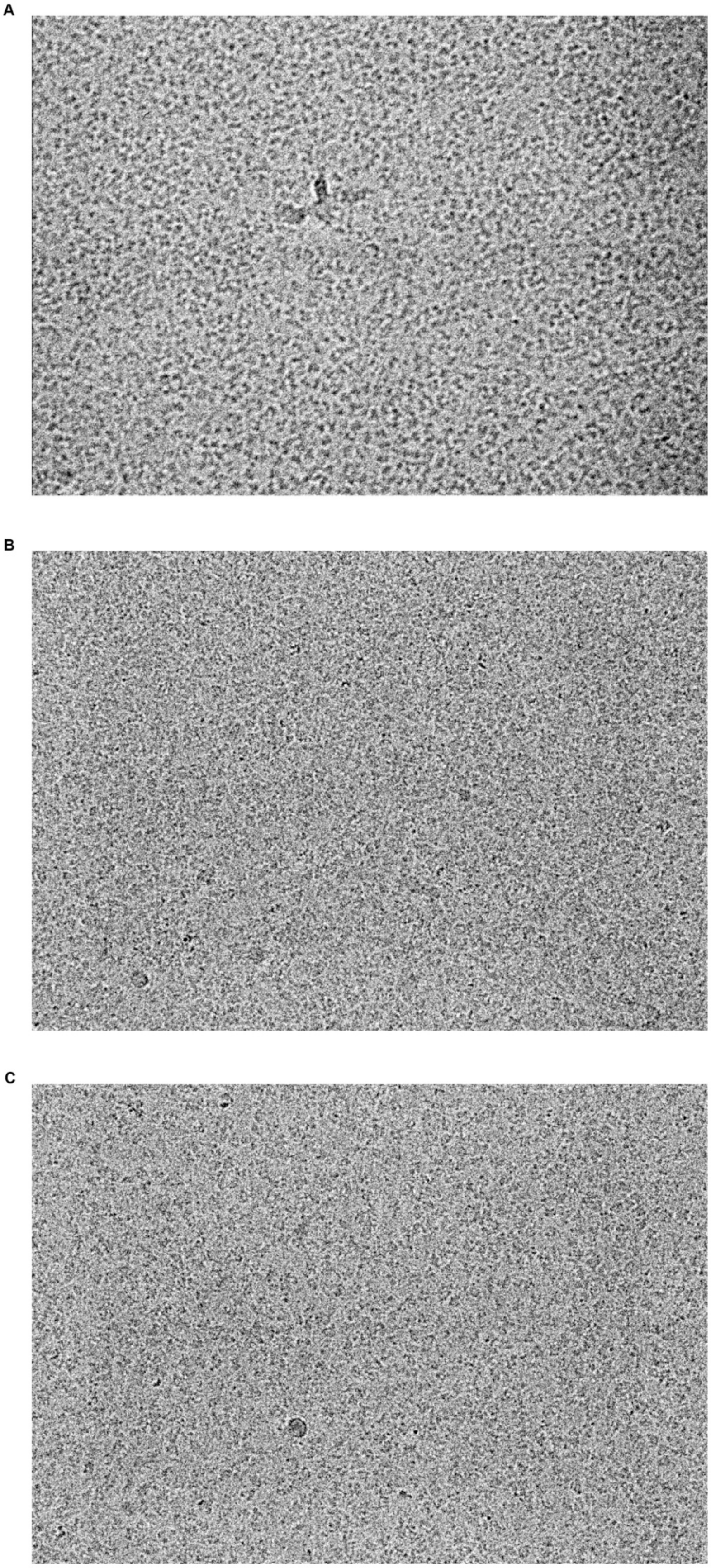
A
B
C

Figure S1. Example micrographs. (**A**) Drift-corrected and summed movie acquired during grid screening at high defocus (approx. 3 µm underfocus). The high defocus helps visualising the distribution of CAK particles in vitreous ice. (**B**, **C**) Drift-corrected and summed movies acquired during data collection for high-resolution structure determination of CAK-ICEC0942 (B, grid 2; C, grid 3) at approx. 1.1-1.2 µm underfocus. The contrast in all three panels has been enhanced to visualise the particles, which are otherwise hard to see due to the low image contrast inherent in cryo-EM data.

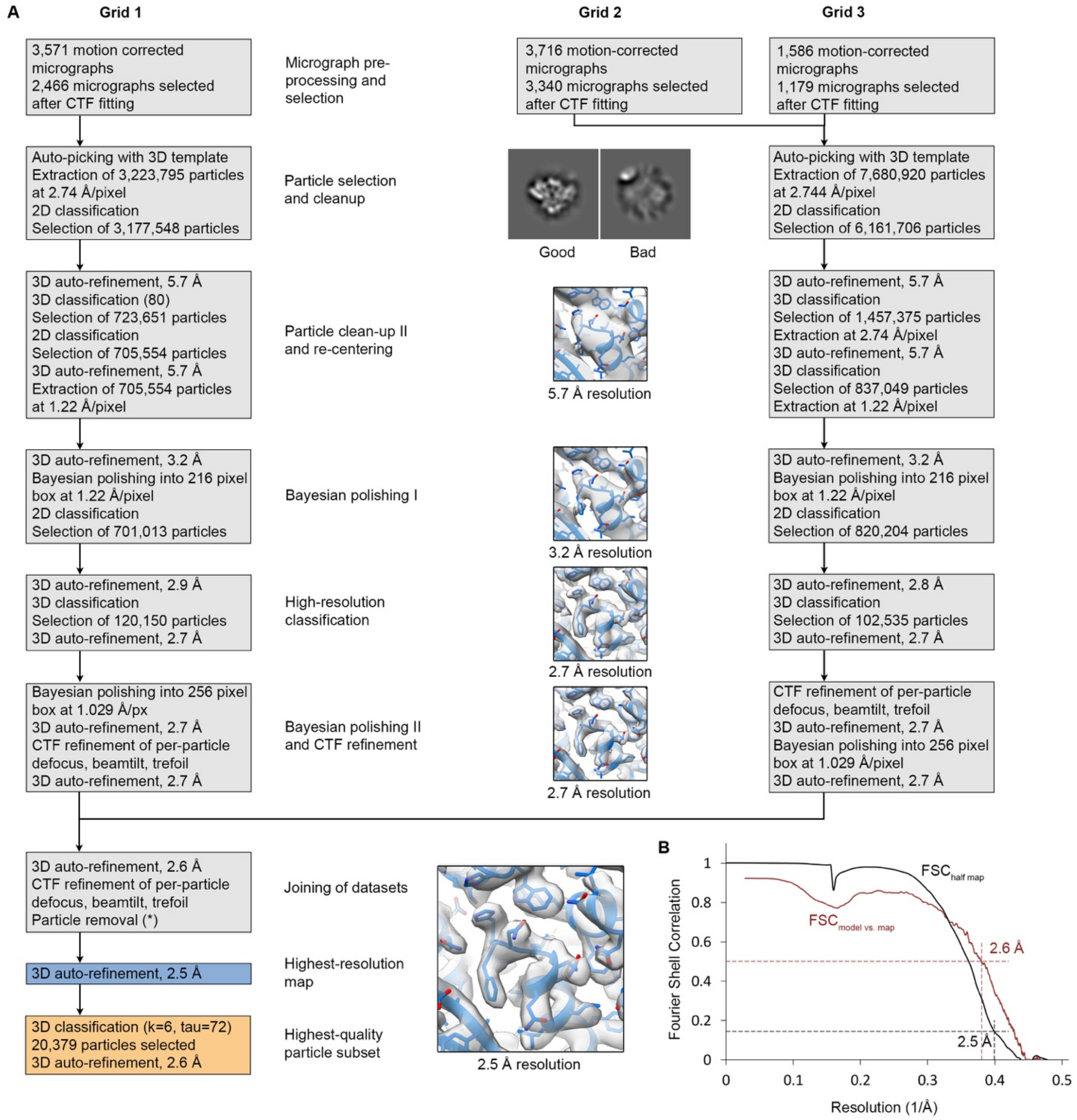

Figure S2. Data processing strategy. (**A**) Individual processing steps are listed, and major phases of processing are outlined. Densities are RELION refinement output maps, shown without B-factor sharpening applied. At the particle removal step (*), particles that came from micrographs with worse than 4 Å estimated CTF fit resolution as well as a very small number of duplicated particles were removed. (**B**) Fourier shell correlation curve computed between cryo-EM half-maps (black) and the map and the refined model (red). Estimated resolutions are indicated.

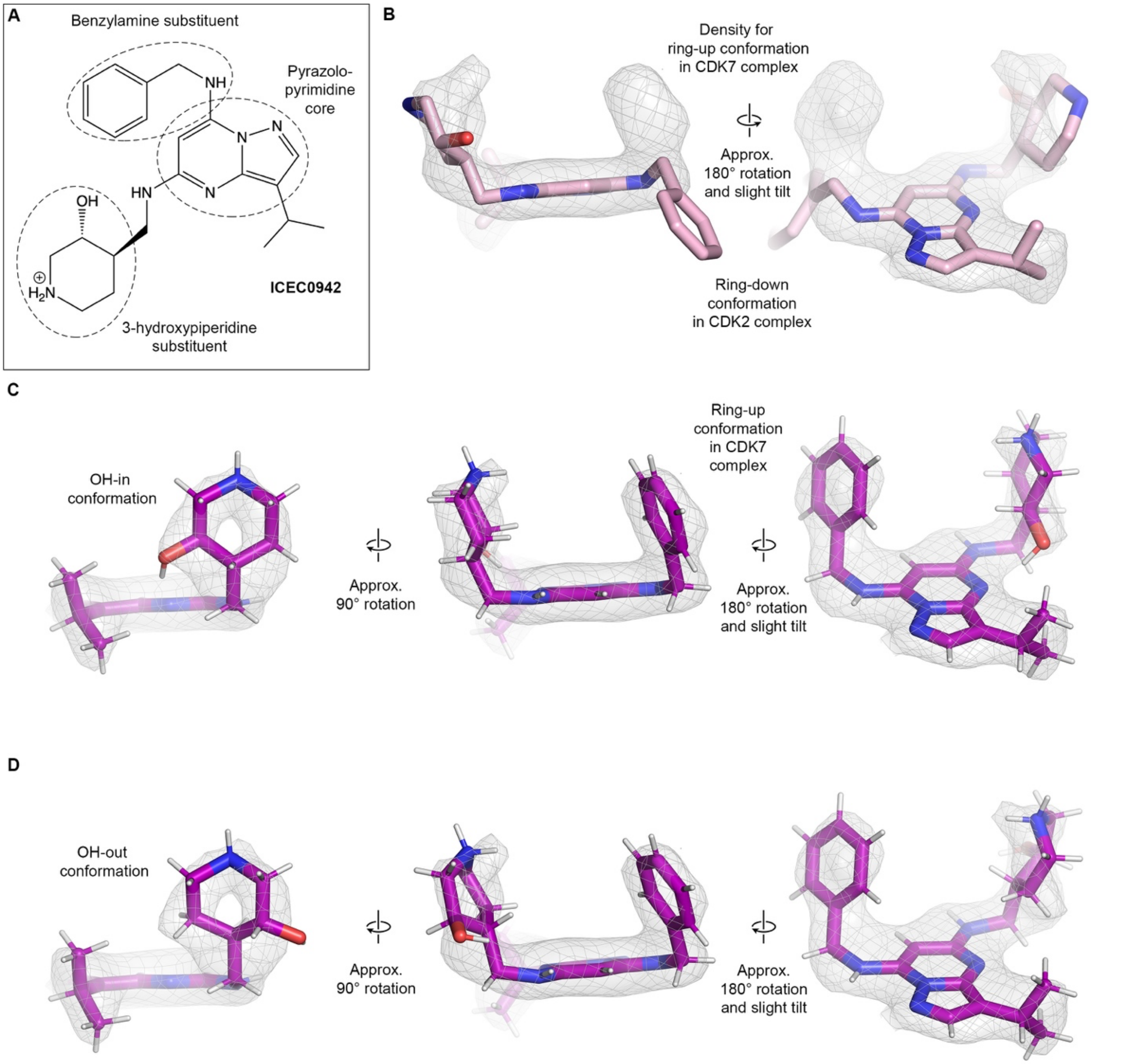

Figure S3. Ligand fitting. (**A**) Chemical structure of ICEC0942. Chemical groups discussed in the text are labelled. (**B**) Comparison of the density for ICEC0942 in complex with CDK7 (density grey) and the structure of the inhibitor as observed bound to CDK2 (pink). The two views are related by a rotation of approx. 180°, with the right hand-side view slightly tilted forward for best visibility of the map fit. (**C**, **D**) Views of ICEC0942 (purple) in the OH-in (C) and OH-out conformations (D) after refinement with PHENIX-OPLS3e, shown with the cryo-EM density (grey). The middle and right-hand side view are the same as in (B). The view on the left is related to the middle panels by a rotation of approx. 90°.

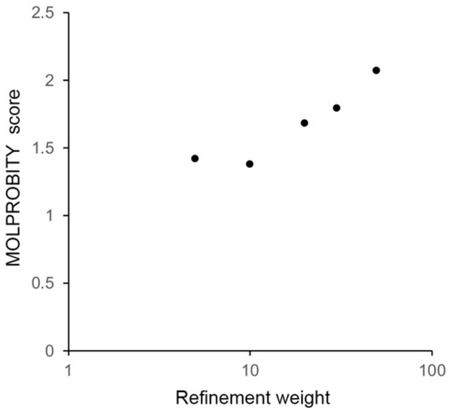

Figure S4. PHENIX-OPLS3e refinement. Due to the higher bond length and bond angle R.M.S.D induced by the use of the OPLS3e force field, an explicit scan across refinement weights in PHENIX was conducted instead of using automated weight determination. Higher weights lead to better fit to the map but worse structure quality as measured by the MOLPROBITY score. We chose 10 as the optimum weight for refinement.
